# Biomarkers in Hereditary Spastic Paraplegias

**DOI:** 10.3390/ijms26051950

**Published:** 2025-02-24

**Authors:** Emanuele Panza, Antonio Orlacchio

**Affiliations:** 1 Dipartimento di Scienze Mediche e Chirurgiche, Università di Bologna, 40138 Bologna, Italy; emanuele.panza@unibo.it; 2 Medical Genetics Unit, IRCCS Azienda Ospedaliero-Universitaria di Bologna, 40138 Bologna, Italy; 3 Dipartimento di Medicina e Chirurgia, Università di Perugia, 06123 Perugia, Italy; 4 Laboratorio di Neurogenetica, Centro Europeo di Ricerca sul Cervello, IRCCS Fondazione Santa Lucia, 00143 Rome, Italy

**Keywords:** hereditary spastic paraplegias, biochemical markers, imaging biomarkers, clinical biomarkers

## Abstract

Hereditary spastic paraplegias (HSPs) represent a group of neurodegenerative disorders characterized by progressive spasticity and weakness in the lower limbs, with no specific treatment available for patients. At the same time, the molecular diagnosis is complicated by the high genetic heterogeneity of this group of diseases, and it can be challenging due to overlapping clinical features with other conditions. Reliable biomarkers could play a fundamental role in diagnosis, prognosis, and therapeutic interventions for HSPs. For this reason, it is necessary to increase the search for biomarkers that can be used to rapidly classify HSPs, follow the natural history of the conditions, and monitor disease correction therapies. This article provides an overview of the current understanding of biomarkers in HSPs, including genetic, biochemical, and clinical biomarkers and new cell imaging-based approaches. In this manuscript, we aim to provide an overview of the current situation in HSP biomarkers, emphasizing the limitations and the necessity of conducting more studies in this field.

## 1. Introduction

Hereditary spastic paraplegias (HSPs) are a genetically and clinically diverse group of neurodegenerative disorders, primarily marked by spasticity and weakness in the lower limbs. These characteristic symptoms can worsen over time, often requiring patients to rely on walking aids or, in severe cases, necessitating the use of a wheelchair [1,2,3]. These conditions can be presented as complicated forms when spastic paraplegia is accompanied by additional neurological or non-neurological symptoms [4].

Taken singularly, HSPs are rare conditions, but when considered as a whole, with more than 80 forms described, they represent an important health issue with a high social and economic impact on the health system [5]. Indeed, depending on the country, the prevalence can vary from 0.3 to 5.5 per 100,000 people, with high socio-economic costs per year [6]. There is an unmet need for specific treatments for HSPs, and therefore, an effort to increase research and clinical trials is highly demanded.

From a diagnostic and prognostic point of view, there is a need to have tools to efficiently classify different HSP forms and follow the evolution of the disease.

Despite the use of neuroimaging techniques [7], pointing to a diagnosis of HSPs remains challenging because of the genetic heterogeneity and overlapping clinical features with other neurological conditions [8].

Developing fast and reliable tests is essential for classifying HSPs and monitoring disease progression effectively. Although identifying a single marker or a few markers to account for all the different forms of HSP is not feasible, advancements in understanding HSP gene products can aid in functionally classifying these disorders based on biological pathways. This progress has the potential to develop more effective treatments that could address multiple forms of HSP simultaneously. In this context, the search and validation of biomarkers are of fundamental importance to assess the effect of such treatments, monitor the development of the disease, and help in rapid classification into specific forms.

In this perspective, we explore the recent development of biomarkers in HSPs, emphasizing their clinical relevance and limitations.

## 2. Main Text

### 2.1. Molecular Testing

The widespread adoption of next-generation sequencing (NGS), including disease gene panels, whole-exome sequencing, and whole-genome sequencing, has transformed genetic analysis. This technology has dramatically enhanced our ability to perform molecular diagnoses by allowing for comprehensive analysis of the entire genome. As a result, geneticists now can achieve molecular diagnoses more rapidly than ever before.

In the case of HSPs, molecular testing serves as the ideal biomarker for distinguishing between various forms of HSPs. It facilitates genetic counseling and, if a genotype–phenotype correlation is established, can predict the natural progression of the disease.

Nevertheless, although more than 80 forms have been described, even with the use of novel sequencing technologies, a molecular diagnosis of HSP patients is reached in only about half of the cases [9]. This can be due to the failure to identify pathogenic variants in causative genes that have yet to be discovered, or because current screening methods are unable to detect causative variants in non-canonical genome regions, such as deep intronic variants that cause aberrant splicing [10,11,12]. For this, it is anticipated that the combined use of omics technologies, such as whole-genome and transcriptome sequencing, will identify and evaluate the effect of deep intronic variants. Even if there are a few exceptions where individuals with mutations in the same gene may exhibit varied symptom progression or even different diseases (e.g., SPG4, SPG9), identifying mutated genes in HSP patients is crucial because these mutations strongly influence the pathology. This understanding is also functional to the comprehension of disease mechanisms.

Nevertheless, the comprehension of pathogenetic mechanisms is not always solely explained by mutations. Reliable, independent biomarkers that can provide insights into the status of a disease and track its progression are also needed. Biomarkers are essential for evaluating the effectiveness of therapeutic interventions and making informed adjustments in patient care to improve disease management. Moreover, applying biomarkers would increase our understanding of the natural history of the disease and could contribute significantly to the development of new treatments and the optimization of existing ones.

### 2.2. Clinical Assessment

Beyond a full medical history record, clinical assessment, and genetic analysis, the introduction of standardized clinical rating scales, such as the Spastic Paraplegia Rating Scale (SPRS) or the Modified Rankin Scale (mRS), enabled objective quantification of spasticity, weakness, gait disturbances, and activities of daily living [13], also giving the opportunity to follow disease progression.

### 2.3. Imaging

Among clinical assessments, neuroimaging techniques such as MRI and positron emission tomography (PET) are widely used diagnostic tools that can provide neurologists with valuable insights into the structural and functional changes associated with neurological conditions, including alterations related to HSPs [14,15].

Recent works show that these techniques have been implemented in different diagnoses applied to HSPs. Quantitative magnetic resonance neurography (MRN), a specialized MRI technique to visualize nerves, showed potential to diagnose HSPs beyond their genetic cause. Recent studies that involved SPG4 and SPG7 patients applied to MRN, and the results supported evidence of peripheral nerve involvement, even without electrographically manifested polyneuropathy. Furthermore, the good correlation of MRN evidence with clinical measures of disease progression supports their use as potential progression biomarkers in HSPs [14]. Moreover, a retrospective qualitative study in SPG76/*CAPN1* patients was able to identify the presence of MRI patterns of white matter abnormalities (periventricular white matter involvement, associated with multifocal subcortical abnormalities, and multifocal subcortical involvement), providing tools to rule out differential diagnosis and diagnose this form of HSP more rapidly [16].

Some specific imaging findings such as the thinning of the *corpus callosum* in SPG11 or SPG15 and the atrophy of the cerebellum in SPG7 are used for diagnosis, even if they are not indicative of disease severity [17,18,19]. In general, spinal cord MRI can reveal common characteristic findings, such as thinning of the spinal cord, particularly in the corticospinal tracts, and white matter abnormalities in the brain. Diffusion tensor imaging (DTI) allows quantitative assessment of microstructural changes in white matter tracts, offering potential biomarkers for disease progression and treatment response [20]. Additionally, PET imaging with radiotracers targeting specific neuronal pathways and cellular processes has been employed to assess clinical signs associated with ATL1 pathogenic variants in SPG3A. PET imaging is also able to detect small-fiber neuropathy, cerebellar hypometabolism, and dysexecutive syndromes associated with the condition [21].

The limitations of using such technologies primarily stem from the rarity of these conditions, which leads to very small sample sizes. Additionally, combining different cases often results in heterogeneous cohorts. Finally, the lack of postmortem validation and the sometimes-limited clinical assessments available are confounding elements for accurate analysis [22]. For these reasons, innovative approaches are essential. Current solutions include robust longitudinal designs, multimodal imaging, and cognitive assessments, which can be applied to study various HSP cohorts or even compared with other motor neuron diseases to identify shared biomarkers and common pathogenetic pathways [23]. Furthermore, collaborative multicenter studies, using harmonized protocols, would address sample size limitations. There is also a need for comprehensive clinical profiling that would be essential to assess clinic–radiological correlations.

### 2.4. Molecular Markers

More molecular/biochemical markers directly related to HSPs were recently identified. This is an interesting field for HSPs that stems from a deeper understanding of the molecular and biochemical causes of these conditions. Among them, a study aimed at analyzing bone homeostasis in 14 SPG5 patients as a non-motor feature found osteopenia in these patients. Furthermore, the authors identified a deficiency in vitamin D3 metabolites (calcidiol and calcitriol), an increase in sclerostin—a factor that inhibits bone formation and mineralization—and a decrease in cross-linked N-telopeptide of type I collagen (NTX), a marker of reduced bone resorption. Additionally, statin treatment was evaluated in samples from the STOP-SPG5 trial, revealing that atorvastatin can normalize elevated sclerostin levels. This finding highlights an additional aspect of osteopenia that complicates the clinical presentation of SPG5. It suggests the need for vitamin D3 supplementation in these patients and positions sclerostin as a potential therapeutic target and novel biomarker for future clinical trials in SPG5 [24].

Other more general markers, such as CSF proteins, have been validated as proxies for neurological damage for different conditions. Neurofilament light chain (NfL) is a structural protein found within large-fiber myelinated axons of the human central and peripheral nervous system and is an established fluid biomarker of neuronal injury [25,26]. Despite its lack of specificity, NfL can be used as a proxy for neurological damage, as it is released into the CSF and bloodstream upon axonal injury, with a direct correlation with disease severity and progression [25,26]. NfL levels as outcome measures are increasingly being used in clinical trials. As evidence, the FDA approval of Tofersen, an antisense oligonucleotide for the treatment of SOD1 amyotrophic lateral sclerosis (ALS), on 25 April 2023 is based on changes in blood NfL levels, outlining a major change in the landscape of what biomarkers mean for regulatory approvals.

The translational use of NfL detection in clinical laboratories for the screening and monitoring of neurodegenerative diseases hinges on its method of measurement. NfL concentration in biofluids is typically assessed using sandwich immunoassays. However, these immunoassays have notable limitations, including the potential for unreliable absolute quantification due to protein modifications and epitope masking. Additionally, they are constrained by the commercial production of antibodies, which hampers characterization and adaptability, and they are relatively expensive, making them unsuitable for widespread clinical application [27].

Recent efforts have been made to promptly profile NfL, developing novel rapid targeted mass spectrometry (MS) assays [28]. These assays for the measurement of this key biomarker in translational neuroscience research can be easily multiplexed and consistently translated into different clinical laboratories for the screening and monitoring of neurodegenerative disease [28]. Standardizing and ensuring cross-compatibility of neurofilament measurements across various analytic platforms are crucial steps for integrating neurofilaments into common clinical practice.

### 2.5. Metabolic Markers

**Figure 1:**
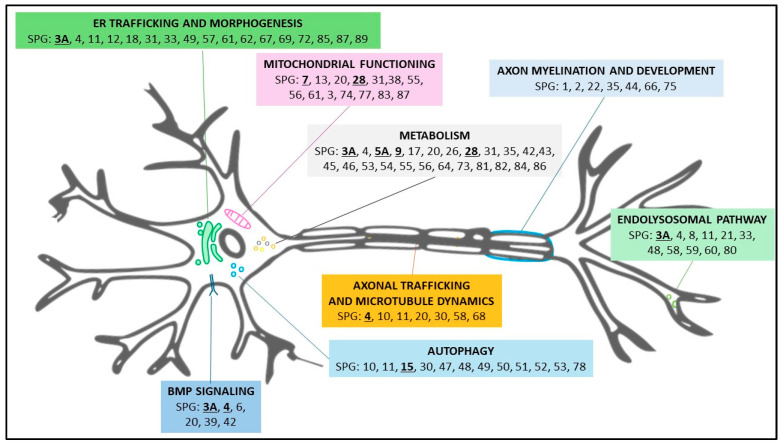
Proposal of aggregation of HSP forms based on gene functions and pathways. Forms described in the text are underlined.

Accessibility and ease of assessment are essential for biomarkers. Ideally, metabolites altered in various forms of metabolic HSPs would serve as optimal biomarkers for analysis. As novel HSP forms are identified and a deeper understanding of the pathogenetic mechanism is reached, novel biochemical biomarkers are discovered (Figure 1).

For instance, oleic acid is a novel blood biomarker for SPG28/*DDHD1* [29]. *DDHD1* encodes the enzyme phospholipase A1, which converts phospholipids to lysophospholipids. Alterations in these lipids could underline SPG28, even in subtle forms. Morikawa and colleagues, studying the lipidome in *Ddhd1*-knockout mice, showed changes in the levels of phosphatidylinositols. Of those phosphatidylinositols, four molecular species with oleic acid dramatically increased in the knockout mice as well as in serum from SPG28 patients. These data indicated that the presence of oleic acid-containing phosphatidylinositols can potentially serve as blood biomarkers for SPG28 following the loss of DDHD1 function [29].

Other biochemical markers are associated with alterations caused by impaired enzymatic activity resulting from mutations in HSP genes. Notably, an emerging area in this field is the study of metabolic HSPs [30]. The identification of mutations in *ALDH18A1* causing the complicated HSP form SPG9 (P5CS deficiency, SPG9A MIM601162; SPG9B, MIM616586) [31,32] pointed toward the identification of a common biochemical pathway where two other well-known Inborn Errors of Metabolism, Hyperornithinemia, Hyperammonemia, and Homocitrullinuria syndrome (HHH, MIM 238970) and arginase deficiency (ARG1 deficiency, MIM 207800), are characterized by a phenotype where spastic paraplegia is present and highly penetrant [33,34,35]. This metabolic pathway involves the metabolism of glutamate connected to the urea cycle (Table 1).

All these conditions are characterized by alterations in plasma amino acids (Table 1). It is relevant to underline that SPG9 can be inherited as a dominant or recessive autosomal condition. As expected, clear-cut altered amino acid levels are particularly evident in recessive forms (SPG9B). Nevertheless, the accessible plasma evaluation of these amino acids represents an easy biomarker to identify this subgroup of HSPs and to distinguish between these three forms.

### 2.6. Potential AI Applications

Recent advances in artificial intelligence (AI), particularly machine learning approaches, have profoundly impacted medical research. Image analysis has greatly benefited from software capable of identifying specific cellular or subcellular patterns. In the context of HSPs, AI holds promising potential for advancing understanding and improving diagnosis through the analysis of various imaging modalities.

An interesting application to the HSP field of biomarkers is represented by computer-assisted deep imaging analysis, in an automated, simple, fast, and non-invasive cell imaging-based method. This kind of visual approach aims to identify patterns of subcellular alterations in affected versus control samples, by enabling the automated segmentation and quantification of key subcellular structures and pathological features in imaging data.

This approach enables the extraction of complex patterns and features from imaging data that may not be immediately apparent to the human eye. In this case, good candidates could be the HSP forms that affect specific organelles such as mitochondria, or the forms that affect the endoplasmic reticulum dynamics (Figure 1). This can facilitate the identification of subtle disease markers and support the development of predictive models for HSP progression. In this way, AI-driven image analysis can contribute to the identification of imaging biomarkers associated with HSP subtypes or disease severity. Custom programs written in commercial software (for example, MetaMorph (version 7.7), ImagePro Plus (7.1), MATLAB (R2024b), or Java(SE 23) have been used to identify, measure, and track cells in images and time-lapse movies [36]. The pattern identified was then used as a specific phenotype that, in turn, can be exploited as a valuable biomarker. Besides these general open-source programs, specific software, such as CellProfiler (4.2.8.), is available to perform this type of analysis [37]. This is an open-source program also widely used by companies, with a large body of literature supporting its applications.

**Figure 2:**
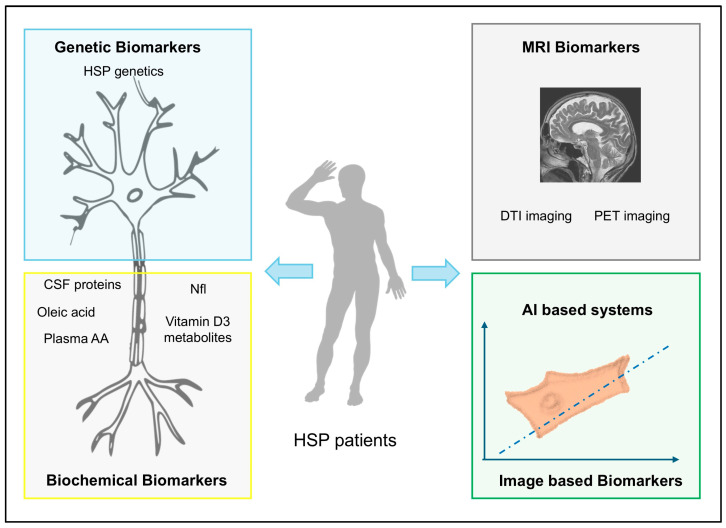
Schematic representation of the array of biomarkers described in HSP.

Recent publications have highlighted specific applications in the field of HSPs. Using this approach, the authors successfully quantified changes in microtubule cytoskeleton organization in patients’ lymphoblastoid cells and peripheral blood mononuclear cells. The method was shown to effectively distinguish SPG4/*SPAST* hereditary spastic paraplegia from both healthy donors and other subtypes of hereditary spastic paraplegia [38]. This demonstrates a rapid, non-invasive, and inexpensive test for recognizing the SPG4 hereditary spastic paraplegia subtype and evaluating the effects of spastin-enhancing drugs in non-neuronal cells (Figure 2).

## 3. Discussion

HSPs represent a very heterogeneous group of rare and ultra-rare diseases. Current clinical classifications are insufficient to describe the complexity of this group of diseases. Genetic analysis made it possible to identify many forms, in some cases described by single cases. We and others previously suggested that classification based on molecular pathways can be more efficient in distinguishing HSP forms with commonly affected pathways [5,8]. This approach is instrumental in identifying molecularly similar forms that could eventually lead to therapies capable of treating more than one HSP form.

In line with this view, it is critical to achieve the following:

1. Characterize the cases that are negative for gene mutation analysis.
2. Better understand the function of HSP gene products.

One possible explanation for HSP patients who test negative in genetic analysis is the lack of studies that combine genome sequencing and transcriptome analysis to identify intronic and deep-intronic causative variants, even in genes already identified. This is a gap that must be filled.

As a complementary approach, it would be ideal to have a range of biomarkers to classify HSPs. In fact, biomarkers play a crucial role in the diagnosis, prognosis, and clinical management of individuals with hereditary spastic paraplegias. These markers could also prove invaluable in assessing therapeutic responses during clinical trials. While numerous markers have been identified in HSP patients, most of them serve diagnostic purposes, with only a limited number currently suitable for predictive use.

While genetic testing is very effective in the characterization of HSP subtypes, there is a great need to have biomarkers to effectively score disease progression. Here, we tried to give an overview and report novel biomarkers and methods to assess disease severity and progression. Despite many efforts, more work needs to be conducted to overcome issues such as the lack of specificity in distinguishing different HSP forms and the challenges related to translation and practicability into the clinical setting. Nonetheless, recent research shows the use of novel approaches applied to the field of HSPs, such as AI-assisted systematic image analysis, to identify altered structures that could be used as novel biomarkers. This is an interesting and emerging field of application that could help in filling the gap of missing biomarkers (Table 2).

## 4. Conclusions

At this moment, it seems necessary to try to integrate biomarker data with clinical assessments to facilitate early diagnosis, monitor disease progression, and evaluate treatment responses in HSPs. Integrating multimodal analysis approaches could advance research, improve diagnostic accuracy, and enable tracking of the effects of targeted therapies for HSPs. One general conclusion that we could draw, while working with such rare and heterogeneous groups of diseases, is the necessity to establish and encourage public efforts to collect cases to generate cohorts of HSP patients. These cohorts could be represented by cases grouped by commonly affected pathways. Thus, collaborative efforts across disciplines are essential for further elucidating the complex mechanisms underlying HSPs. Future research efforts should help focus on validating and standardizing biomarkers across different HSP subtypes and exploring their potential as therapeutic targets for disease-modifying interventions.

This has the final aim of translating biomarker discoveries into effective clinical practice, and it will eventually lead to more accurate diagnosis and prognosis, but especially to more specific and personalized treatment strategies for individuals affected by HSP.

## Figures and Tables

**Table 1:** Biochemical alterations in P5CS deficiency, ARG1 deficiency, and HHH syndrome.

		P5CS Deficiency	HHH Syndrome	ARG1 Deficiency
	Disease Inheritance	AR AD	AR	AR
Plasma ammonia		↑± (fasting)	↑+ (fed)	↑+ (fed)
Plasma ornithine		↓±	↑+	–
Plasma arginine		↓±	–	↑+
Plasma citrulline		↓±	↓±	–
Plasma proline		↓±	–	–
Homocitrullinuria		–	+	–
Orotic aciduria		–	+	+

↓± = Reduced levels in most of the cases; ↑± = Increased levels in most of the cases; ↑+ = Increased levels in all cases; + = characteristic feature; – = absent feature.

**Table 2:** Schematic representation of HSP biomarkers and technologies discussed in the text.

Biomarkers	Target	Technology	HSP Forms
Molecular Testing	HSP genes	WES, WGS, gene panels	all
Imaging		PET, MRI, MRN, DTI	Commonly used for general assessment. In the text, we report uses for SPG3A, SPG4, SPG7, SPG11, SPG15, and SPG76
Molecular markers	Vitamin D3 metabolites, statin, NfL	Biochemical assays	SPG5 and general marker for neurodegenerative disease
Metabolic markers	Plasma amino acid concentration	Custom enzymatic tests, mass spectrometry	SPG9A, SPG9B, SPG28
AI application	Cell features	Cell Imaging coupled with software	SPG4

## Data Availability

Not applicable.
